# Metabolite Profiling of Paraquat Tolerant *Arabidopsis thaliana Radical-induced Cell Death1* (*rcd1*)—A Mediator of Antioxidant Defence Mechanisms

**DOI:** 10.3390/antiox11102034

**Published:** 2022-10-15

**Authors:** Nina Sipari, Jenna Lihavainen, Markku Keinänen

**Affiliations:** 1 Viikki Metabolomics Unit, Organismal and Evolutionary Biology Research Programme, Faculty of Biological and Environmental Sciences, University of Helsinki, P.O. Box 65, FI-00014 Helsinki, Finland; 2 Department of Environmental and Biological Sciences, University of Eastern Finland, P.O. Box 111, FI-80101 Joensuu, Finland; 3 Umeå Plant Science Center, Department of Plant Physiology, Umeå Universitet, 90 187 Umeå, Sweden; 4 Institute of Photonics, University of Eastern Finland, P.O. Box 111, FI-80101 Joensuu, Finland

**Keywords:** *Arabidopsis thaliana*, *rcd1*, ROS, paraquat, metabolite profiling, antioxidants, LC−MS, GC−MS, ascorbate, glutathione

## Abstract

RADICAL-INDUCED CELL DEATH1 (RCD1) is an *Arabidopsis thaliana* nuclear protein that is disrupted during oxidative stress. RCD1 is considered an important integrative node in development and stress responses, and the *rcd1* plants have several phenotypes and altered resistance to a variety of abiotic and biotic stresses. One of the phenotypes of *rcd1* is resistance to the herbicide paraquat, but the mechanisms behind it are unknown. Paraquat causes a rapid burst of reactive oxygen species (ROS) initially in the chloroplast. We performed multi-platform metabolomic analyses in wild type Col-0 and paraquat resistant *rcd1* plants to identify pathways conveying resistance and the function of RCD1 in this respect. Wild type and *rcd1* plants were clearly distinguished by their abundance of antioxidants and specialized metabolites and their responses to paraquat. The lack of response in *rcd1* suggested constitutively active defense against ROS via elevated flavonoid, glutathione, β-carotene, and tocopherol levels, whereas its ascorbic acid levels were compromised under non-stressed control conditions when compared to Col-0. We propose that RCD1 acts as a hub that maintains basal antioxidant system, and its inactivation induces defense responses by enhancing the biosynthesis and redox cycling of low molecular weight antioxidants and specialized metabolites with profound antioxidant activities alleviating oxidative stress.

## 1. Introduction

Paraquat (methyl viologen, MV, 1,1′-dimethyl [4,4′-bipyridine]-1,1′-diium dichloride) is a widely used herbicide that is highly toxic for plants due to its ability to form highly reactive oxygen species (ROS). ROS are also byproducts of normal metabolism of oxygen—they act as signaling molecules, but in excess, they can cause oxidative damage that may ultimately lead to cell death. In plants, MV causes rapid membrane damage by accepting electrons from Photosystem I (PSI) and subsequently transferring them to molecular oxygen, resulting in the increased production of ROS, initially in chloroplasts, which efficiently induce cell death [1]. In addition, MV exposure enhances the linear photosynthetic electron transport rate (ETR), increases the pH gradient (∆pH) formation across the thylakoid membranes in chloroplasts, and decreases NADPH levels, providing conditions for the de-epoxidation of the xanthophyll cycle and resulting in increased levels of zeaxanthin (Zx) and antheraxanthin (Ax) [2,3,4,5]. Superoxide dismutase (SOD) is the main antioxidative enzyme in chloroplast that converts superoxide to hydrogen peroxide (H_2_O_2_), which is further scavenged by the antioxidant system or translocated to other cell compartments to be scavenged [6,7,8]. Because of its widespread and repeated usage, MV resistance has naturally evolved in various weeds due to mutations that either limit the uptake or confer tolerance via other mechanisms such as sequestration, detoxification via active metabolism, decreased translocation to chloroplasts, or with enhanced ROS scavenging ability by enzymatic antioxidants [9].

*Arabidopsis thaliana rcd1* (*radical-induced cell death1*) is highly resistant to MV-induced chloroplastic ROS but sensitive to ozone and apoplastic superoxide [10,11,12]. Even in control conditions in light, superoxide accumulates in *rcd1* plants, but the known metabolic oxidative stress markers [13] or the transcripts connected to oxidative stress (e.g., enzymatic antioxidant) and/or programmed cell death (PCD) are not deregulated in *rcd1* [14,15]. This makes the *rcd1* an interesting mutant to study redox and antioxidative metabolism as it is resistant and susceptible against different stressors. In addition to MV resistance, plants that have impaired function of RCD1 have several other phenotypes beyond the altered sensitivity to ROS [10,11,12,16]. They exhibit stunted growth, altered leaf and rosette morphology, defected root development, more open stomata, early flowering and early senescence, and altered response to phytohormones and nitric oxide (NO) [10,16,17,18,19,20]. Other stress-related phenotypes of *rcd1* include tolerance to glucose, mannitol, freezing and UV-B radiation, and sensitivity to salt [16,17,19,20,21,22]. Even though these phenotypes of *rcd1* are known, the underlying mechanisms remain unclear. RCD1 has also several interaction partners (e.g., ANAC017 and -103, DREB2A, STO), and it inhibits the expression of several genes and transcription factors connected to stress, including mitochondrion dysfunction stimulon (MDS) genes (e.g., alternative oxidases AOXs, SOT12) [18,23]. In addition, RCD1 inhibits STO (SALT TOLERANCE) and COP1 (CONSTITUTIVELY PHOTOMORPHOGENIC1)-regulated genes after UV-B radiation [21,24,25], and the interaction with STO and COP1 represses the *ELONGATED HYPOCOTYL5* (*HY5*) transcription. HY5 is a major regulator of light and temperature responses in plants, and it mediates the biosynthesis of various specialized (secondary) metabolites (e.g., flavonoids, glucosinolates) [21,24,25].

Plants produce numerous structurally and functionally diverse specialized metabolites that play different roles in plant growth and development, as well as function as antioxidants and convey defense against abiotic and biotic stresses. The hydroxycinnamic acids and monolignol/lignans glycosides act as building blocks and precursors for lignin biosynthesis affecting the structure of cell wall as well as growth of the whole plant [26], and the changes in the lignin biosynthesis are a result of balancing growth and development, and defense responses [27]. Flavonoids are one of the major secondary metabolite groups in plants [28]. They act as UV filters and in defense against pathogens, but they also have a key role in coloration, nodulation, and pollen fertility [28]. The regulation of the phenylpropanoid pathway as well as the modification reactions involve numerous transcription factors (e.g., MYBs and NACs) and several UDP-glucuronosyltransferases (UGTs) and sulfotransferases (SOTs/SULTs) [29,30,31]. The glycosylation of several specialized metabolite groups (e.g., phenylpropanoids, phytohormones, and monolignols/lignans), increase their water solubility, stability, and bioavailability, affecting their compartmentalization and biological activity, and link the biosynthetic routes to sugar metabolism [26,29,30,32,33].

The re-routing of carbon flow from anabolic to catabolic processes during oxidative stress conditions and from growth to defense, as well as activating or rebalancing the antioxidant machinery, is a complex network and is highly regulated in plants. Non-enzymatic antioxidant system consists of low molecular weight metabolites, and it protects the cells from the oxidative damage caused by reactive oxygen and nitrogen species (ROS, RNS), and organic radicals (e.g., lipids) [34,35,36]. The non-enzymatic antioxidants can be divided into hydrophilic and hydrophobic antioxidants. Ascorbic acid (Asc, DHA), glutathione (GSH, GSSG), glycosylated flavonoids, xanthine, and urate are hydrophilic antioxidants, which scavenge ROS/RNS in extra- and intracellular fluids. Even though glutathione and ascorbate are both key players in the Foyer–Halliwell–Asada cycle (i.e., ascorbate-glutathione pathway), their metabolisms are differentially affected by environmental factors [36,37]. Glutathione biosynthesis occurs both in cytosol and chloroplasts from amino acids, while ascorbate synthesis is dependent on sugar metabolism and linked to mitochondrial electron transport chain (ETC) between complexes II and IV (COX) [38,39]. Ascorbate is required as a reducing agent or as a co-factor for a range of enzymes involved in the biosynthesis of specialized metabolites [40], in de-epoxidation reactions in the violaxanthin cycle [41,42] and in the regeneration of tocopherols [43]. It has been reported to be essential for plant growth but not associated with photoprotection [43]. Tocopherols, carotenoids, flavonoid aglycones, and ubiquinols are considered lipophilic/hydrophobic antioxidants, which scavenge mainly organic radicals in hydrophobic membranes [44]. Tocopherols and carotenoids are mainly localized in chloroplast membrane, protecting photosynthetic apparatus against photooxidative stress [45]. Tocopherols quench singlet oxygen radicals and/or chemically scavenge singlet oxygen or lipid peroxyls [45]. They can also act as a membrane stabilizer and regulate the membrane fluidity and integrity [45]. Phylloquinone (vitamin K1) does not function as an antioxidant *per se* but acts as an electron carrier in photosystem I (PSI) [46,47]. Carotenoids, on the other hand, have a role in photoprotection but are also essential in oxygenic photosynthesis by stabilizing the pigment-protein complexes and by harvesting sunlight [48]. For instance, the β-carotene levels in Arabidopsis increase during high light conditions (i.e., plastidial ROS) [49] but also when the flow from xanthophylls to apocarotenoid glucosides is blocked in the biosynthetic mutant plants (*ccd4; carotenoid cleavage dioxygenase 4*) [50], indicating the close regulation of carotene and xanthophyll biosynthesis.

In this study, multi-platform untargeted metabolite profiling was performed to study the general responses to MV, as well as the metabolic features of MV resistant *rcd1* mutant in light and after oxidative stress conditions induced by MV. The metabolite profiles of moderately MV resistant (Col-0) and MV resistant (*rcd1*) plant lines were compared to elucidate the mechanisms of tolerance and the possible functions of RCD1 in this respect. We also discuss how the observed adjustments of specialized metabolic pathways and redox metabolism can define plant defense and development in response to oxidative stress.

## 2. Materials and Methods

### 2.1. Plant Material

Plant material and experimental setup were as described in Sipari et al. [13]. In short, Col-0 (wild type) and *rcd1* (*rcd1-4*, GK-229D11) *Arabidopsis thaliana* seedlings were exposed to light (L) and methyl viologen (MV). Hydrophilic secondary metabolites were analyzed from the same samples as GC-MS metabolites in Sipari et al. [13], and the plant material used for lipophilic secondary metabolite (i.e., carotenoids, tocopherols) analysis was from a replicated experiment.

### 2.2. Extraction of Lipophilic Metabolites

Lyophilized plant material (Col-0 wild type and *rcd1* mutant seedling, *n* = 5) of Arabidopsis (100 mg) was ground into a fine powder with a ball mill (Qiagen TissueLyser II, Qiagen, Germany). Carotenoid extraction was carried out in 2.0 mL centrifuge tubes on ice/in cold room (4 °C), in dark or under dim light. A volume of 700 µL of 30% methanol (LC-MS Chromasolv grade, Sigma-Aldrich, Steinheim, Germany) with 1 mL of chloroform (Sigma-Aldrich, Steinheim, Germany) was added, and the suspension was vortexed for 30 min at 4 °C in dark. Samples were centrifuged at 4 °C in 15 000 rpm (21,500× *g*) for 5 min. The nonpolar hypophase was removed, and the aqueous epiphase re-extracted with chloroform. The pooled chloroform extracts were dried in vacuum concentrator at 45 °C (Genevac, miVac, Ipswich, England). The dried residues were protected from light and stored under nitrogen atmosphere at −80 °C prior to UPLC-PDA-HDMS analysis.

### 2.3. Analysis of Lipophilic Metabolites

The analysis of lipophilic metabolites (carotenoids and tocopherols) was carried out with Waters Acquity UPLC system connected with PDA detector and Waters Synapt G2 HDMS/QTOF mass spectrometer (Waters, Milford MA, USA). Samples were redissolved in 100µL of ACN:MeOH (7:3) and analyzed with ESI ion source using positive ionization mode. Metabolites were separated in Acquity UPLC BEH C18 column (1.7 µm, 150 × 2.1 mm; Waters, Ireland). The injection volume was 2 µL, oven temperature was 30 °C, and sample tray temperature was 25 °C. The chromatographic method was adapted from Fraser et al. [51], Rivera et al. [52], and Gupta et al. [53]. The mass range (*m*/*z*) was from 100 to 1000 and the wavelength range was 220–500 nm. The mobile phase A consisted of methanol/water mixture (98:2), and solvent B of acetonitrile (Chromasolv grade, Sigma-Aldrich, Steinheim, Germany). The linear gradient started with 20% A, changed to 100% B in 15 min, remained at 100% for 10 min, switched back to 20% A and equilibrated for 2 min, with a total analysis time of 27 min. The flow rate of the mobile phase was 0.5 mL min^−1^ for the first 18 min and 0.6 mL min^−1^ for 4 min after which it switched back to 0.5 mL min^−1^. Peak picking from MS-data was executed with MassLynx V4.2 (Waters Corporation, Milford Massachusetts, USA). Carotenoid and xanthophyll peaks were integrated manually from PDA-detector using wavelength of 460 nm. Metabolites were identified by comparing the retention time, UV-spectra, and the exact mass of commercial standards and/or previously published data [51,54]. The data were normalized by the fresh weight (FW, mg) of the sample and de-epoxidation state (DEPS) of xanthophylls was calculated by using the amounts of violaxanthin (Vx), *trans*-zeaxanthin (Zx) and antheraxanthin (Ax) in a formula: (0.5 × Ax + Zx) × (Ax + Vx + Zx)^−1^ [42].

### 2.4. Analysis of Specialized Metabolites

Specialized metabolites were extracted as described in Sipari et al. [13] and analyzed with the same instrumentation as the lipophilic metabolites. Samples were analyzed both in positive (ESI +) and negative (ESI −) ionization mode. The mass range (*m*/*z*) was set to 100–1500 in positive and 100–1000 in negative mode. The compounds were separated on an Acquity UPLC BEH C18 column (1.7 µm, 50 × 2.1 mm, Waters, Ireland). The injection volume was 2 µL, oven temperature was 40 °C, and tray temperature was 10 °C. The mobile phases consisted of (A) H_2_O and (B) acetonitrile (Chromasolv grade, Sigma-Aldrich, Steinheim, Germany) both containing 0.1% formic acid (Sigma-Aldrich, Steinheim, Germany). A linear gradient started from 95% A and proceeded to 10% in 5 min. The eluent composition was changed back to 95% at 5.1 min and left to equilibrate for 0.9 min, with a total analysis time of 6 min. Data processing with MassLynx and the identification of metabolites were performed as described for lipophilic metabolites. The relative levels of the specialized metabolites were calculated by normalizing the analyte peak area by the peak area of the internal standard, 4-methyl-umbelliferone, and the fresh weight of the sample.

### 2.5. Gas Chromatography–Mass Spectrometry

The results of GC-MS analysis have been previously published in Sipari et al. [13]. Here the data from different analysis platforms were merged to provide comprehensive view of the metabolite responses including antioxidants and specialized metabolites, some of which can be detected with both GC-MS and UPLC-MS methods, while other metabolites are detected with one particular method.

### 2.6. Statistical Analysis

Principal component analysis (PCA) was performed with Simca P+ (version 16, Umetrics, Umeå, Sweden) to visualize general variation in the data sets. Metabolites present in at least in one sample group were included in the analyses (missing value threshold of 80%). The analyses were performed with log10-transformed data scaled by unit variance and tested for normality and homogeneity of variance. The main effects of plant line (L), treatment (T, light [L] and methyl viologen [MV]), and line × treatment (L × T) interactions on metabolite levels and metabolite ratios analyzed with different platforms were tested with two-way ANOVA (MetaboAnalyst) [55]. False discovery rate (FDR) was applied for multiple comparisons of individual metabolite levels. In all statistical tests, *p*-value < 0.05 was considered significant and missing values were imputed with probabilistic PCA (PPCA) method (MetaboAnalyst). Pairwise comparisons for line effects in L and MV treatments and the effect of MV within each line were tested with t-tests and visualized in volcano plots (MetaboAnalyst). The number of metabolites with significantly different levels between lines and treatments were then visualized in Venn diagrams (http://www.interactivenn.net/ [56]).

## 3. Results and Discussion

### 3.1. Specialized Metabolism Is Consistently Altered in rcd1

Methyl viologen (MV) is highly toxic to plants as it causes a rapid burst of ROS in the chloroplast that can trigger cell death. Plants have developed different mechanisms to tolerate MV ranging from restricted uptake to efficient detoxification by antioxidant systems. The *rcd1* is among the *Arabidopsis thaliana* mutants that exhibit high MV resistance; however, the mechanisms behind it remain unclear. Thus, we studied and compared the metabolite responses of *rcd1* and wild type Col-0 plants that display moderate MV resistance to elucidate the potential metabolic adjustments providing MV tolerance and the possible functions of RCD1 in respect to the regulation of redox metabolism and specialized metabolic pathways. Our comprehensive analysis included key antioxidants such as glutathione; ascorbate and its catabolites; specialized metabolites (and their precursors) such as hydroxycinnamic acids, flavonoids, and glucosinolates; and lipophilic metabolites such as carotenoids, tocopherols, and other prenyllipids (Supplementary Table S1a–h).

**Figure 1:**
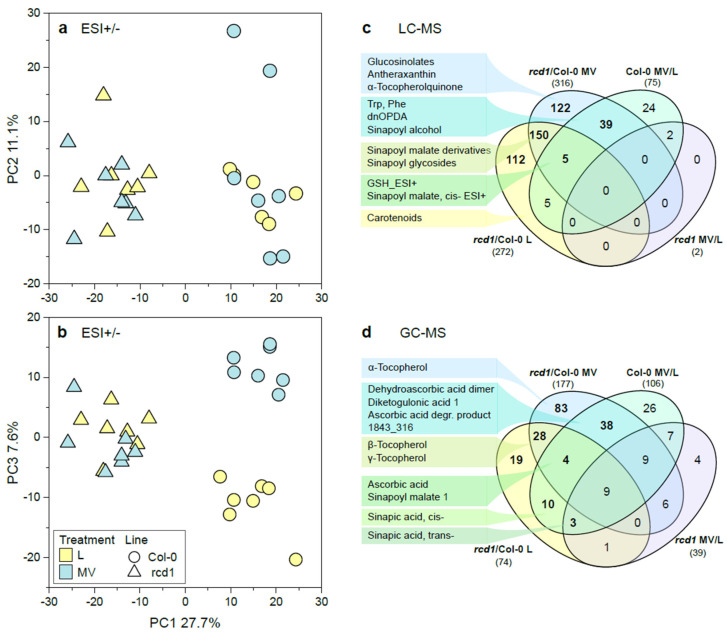
Metabolite profiles in Arabidopsis as affected by line and treatments. Metabolites were analyzed by LCMS with positive and negative ESI, and the variation in metabolite profiles (1146 features, *n* = 7) was studied with principal component analysis (PCA). (**a**) PC1 vs. PC2, (**b**) PC1 vs. PC3. In addition, the variation in lipophilic metabolite profile (16 metabolites analyzed with LCMS using positive ESI) was studied (Supplementary Figure S1d). Venn diagrams (**c**,**d**) depict the overlap of significantly affected metabolites between lines and different treatments. (**c**) Metabolites identified by LC-MS, and (**d**) by GCMS [13] in selected sectors are shown. See the details of statistical results, additional PCA plots, and pairwise comparisons for metabolite levels in Supplementary Figures S1 and S2 and in Supplemental Data set Table S1i–k. L = Light, MV = methyl viologen/paraquat.

The visual phenotypes of two-week-old seedlings of *rcd1* mutant and wild type Col-0 plants did not differ at the time of sampling [13]. However, the metabolic profiles of plant lines were clearly different as observed in principal component analysis (PCA) of primary [13] and secondary metabolite profiles (the first principal component PC1, Figure 1a,b, Supplementary Figure S1). These results demonstrated that not only the primary metabolism (i.e., sugars, sugar phosphates, organic acids, and the majority of amino acids) [13] was altered, but also the specialized secondary metabolite levels were consistently different between *rcd1* and the wild type, Col-0 (Figure 1a–d, Supplementary Figure S2a–d). Approximately 70% (in both L and MV) of significantly changed specialized metabolites were elevated in *rcd1* when compared to Col-0. This indicated that RCD1 acts mainly as an inhibitor of several biosynthetic pathways, and the upregulated, excess carbon flow in primary metabolic pathways (e.g., sugars, amino acids) [13,23] is likely directed to the production of specialized metabolites involved in defense responses, and not to protein synthesis and growth, as manifested by the high levels of various metabolites derived from phenylpropanoid pathway and the dwarf-like phenotype of *rcd1* [57,58,59]. Autoimmune mutants with constitutive defense responses often have reduced growth to different extents compared with the wild type, and those growth defects can be reduced or reverted by inhibition of defense responses [15,60].

### 3.2. Paraquat Exposure has Almost no Effect on Specialized Metabolism in rcd1

Unlike primary metabolism that displayed clear shifts in response to MV treatment irrespective of plant line [13] (Figure 1d), specialized metabolite profile was affected by MV treatment only in Col-0, but not in *rcd1* (Figure 1). In Col-0, the levels of 75 metabolites (LC-MS) were significantly altered after MV exposure, whereas only the levels of two unknown metabolites were similarly depleted by MV in *rcd1* (Figure 1c, Supplementary Figure S1, Table S1j–k). This clearly indicated that the response to MV treatment was much weaker in *rcd1* than in Col-0, especially in terms of specialized metabolism.

### 3.3. RCD1—The Mediator of Phenylpropanoid Pathway and Lignin Metabolism

The differences between Col-0 wild type and *rcd1* lines were seen in the levels of phenylpropanoids such as flavonoids (kaempferol and quercetin glycosides), monolignol (p-coumaryl/p-hydroxyphenyl, coniferyl/guaiacyl, sinapoyl/syringyl alcohol) and lignan (pinoresinol, malylsesamolinol, sesamolinol, lariciresinol, secoisolariciresinol) derivatives (e.g., glucosides or hydroxycinnamic acid esters) (Supplementary Table S1i–k). Phenylpropanoids all originate from shikimate route/phenylpropanoid pathway via same precursors of aromatic amino acids. Flavonoids, monolignols/lignans, and other phenylpropanoids are also connected to sugar metabolism, as majority of them are glycosylated. The levels of shikimate (precursor), sinapate, and metabolites connected to lignin biosynthesis, including unglycosylated phenylpropanoids, e.g., sinapoyl malate, and lignans, their common esters (lignan/oligolignol phenylpropanoid ester), malyl sesamolinol, guaiacylglycerol sinapate as well as feruloyl- and syringaresinol glucoside were systematically lower in *rcd1* than in Col-0 irrespective of treatment (in L and MV) (Figure 1, Supplementary Table S1i–k). Lignin is a heterogeneous polymer, which consists of monolignols and is polymerized at the surface of the cell walls. In addition to monolignols, monolignol glucosides (MLG) have been reported to act as intermediates of lignin biosynthesis [26,61]. The decreased levels of several monolignols and lignin-associated metabolites indicated inhibited lignin pathway in *rcd1* in line with its dwarf-like phenotype [57,58,59]. Thus, we propose that under control non-stressed conditions, RCD1 may direct phenylpropanoid pathway towards lignin biosynthesis to sustain growth.

**Figure 2:**
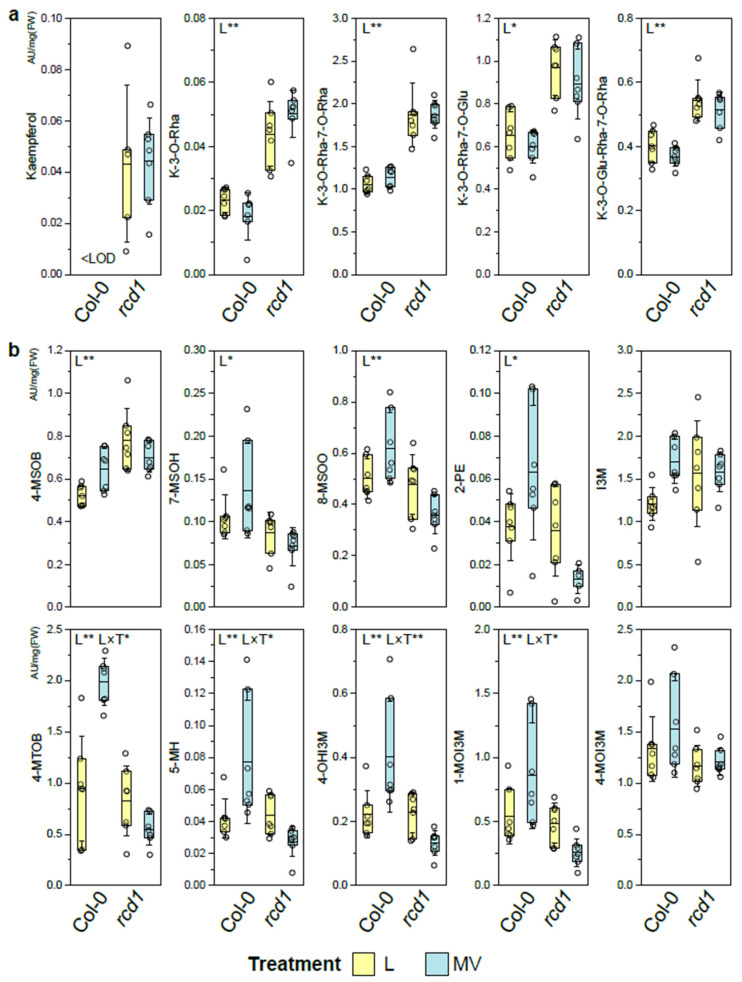
The levels of (**a**) flavonoids and (**b**) glucosinolates in *Arabidopsis thaliana* Col-0 wild type and in *rcd1* mutant plants in control/light conditions (Ctrl/L) and after methyl viologen (MV) treatment. The main effects of plant line (L) and treatment (T) and their interaction (L × T) were tested with two-way ANOVA (FDR-adjusted *p*-value < 0.05 *, <0.01 **); the details of the statistical results are in the supplementary Table S1i. Dots represent data points (*n* = 4–7); the box represents 25 and 75 percentiles; the line marks the mean and the whiskers the standard deviation. The levels are arbitrary units (AU/mg [FW]), peak area normalized with internal standard, and samples fresh weight (FW). The metabolite levels below limit of detection are marked as <LOD. K = kaempferol, Rha = rhamnoside, Glu = glucoside, 4-MSOB = 4-methylsulfinylbutyl-, 7-MSOH = 7-methylsulfinylheptyl-, 8-MSOO = 8-methylsulfinyloctyl-, 2-PE = 2-phenylethyl-, I3M = indolyl-3-methyl-, 4-MTOB = 4-methylthiobutyl-, 5-MH = 5-methylhexyl-, 4-OHI3M = 4-hydroxy-indolyl-3-methyl-, 1-MOI3M = 1-methoxy-indolyl-3-methyl-, 4-MOI3M = 4-methoxy-indolyl-3-methyl glucosinolate (GLS).

The flavonoid levels did not respond to MV on either line and were significantly higher in *rcd1,* irrespective of the conditions (Figure 2a). This can be an outcome of lack of inhibition of *HY5* transcription factor by RCD1. Both RCD1 protein as well as flavonoids have been reported to accumulate in guard cells [18,62,63,64]. The *rcd1* has more open stomata, which are proposed to be co-regulated at least partially by flavonoids [62,64]. Flavonoid accumulation significantly increases tolerance to oxidative stress in various plant species, and their consistently elevated levels can explain several stress phenotypes such as UV-resistance of *rcd1* plants [33,65]. Glycosylation of metabolites is mainly regulated by various UDP-glucosyltransferases (UGTs) [29,66,67,68,69] that glycosylate a wide range of specialized metabolites and are involved in redox homeostasis and responses to various stress factors [33,61]. The surplus of carbohydrates and uridine nucleotides [13,23] may drive the consistently enhanced glycosylation of specialized metabolites (e.g., major flavonoids, caffeoyl-, sinapoyl, and feruloyl bissinapoyl glucosides) observed here in the *rcd1* plants (Figure 2, Supplementary Table S1i–k).

Unlike flavonoids, the levels of most glucosinolates (GLS) did not differ significantly between plant lines in control conditions, but the response to MV differed between Col-0 and *rcd1* (Figure 2b). The majority of GLS levels increased in Col-0 in response to MV, while there was not change or they were depleted in *rcd1* (Figure 2b). Although, some of the glucosinolates (GLS) (i.e., aliphatic GLS) are not synthesized from shikimate route metabolites (Phe, Tyr, and Trp), the biosynthesis of different types (i.e., aromatic, indole) of GLS occur through aromatic amino acids, and GLS biosynthesis is tightly connected to phenylpropanoid and flavonoid biosynthesis [70,71,72]. The GLS biosynthesis has been reported to be well-coordinated (e.g., similar response to sulfur deficiency) and regulated by light and at least partly by (e.g., primary sulfur assimilation) *HY5* [73]. There are also differences in light responses between different MYBs, CYPs, and SOTs (sulphotransferase) in aliphatic, indolic, and aromatic GLS pathways: HY5 acts as a repressor of the MYB factors and an activator for the two pathways of aliphatic and indolic GLS [73]. GLS and flavonoid biosynthesis also share some UGTs, which are involved in GLS biosynthesis but also phenylpropanoid glycosylation and auxin deactivation [74]. Therefore, the crosstalk between the two biosynthetic pathways, GLS and phenylpropanoid pathway, is intertwined and regulated in a complex manner by IAOx and auxin (IAA), by different TFs (via HY5), and possibly by RCD1 during oxidative stress [70,71].

During oxidative stress, the levels of several specialized metabolites including phenolics, carotenoids, and tocopherols increase to re-equilibrate redox balance against increased ROS production and disturbance in the cellular redox state [33,75,76,77]. Consistent with the accumulation of shikimate route metabolites, phenylalanine ammonia-lyase (PAL) is strongly induced at both transcriptional and enzyme activity levels [75,78] similarly to other genes involved in flavonoids biosynthesis [79]. The (direct and secondary) regulation of phenylpropanoid and flavonoid biosynthetic pathways and their modification/glycosylation is highly complex, under the control of numerous TFs, and intertwined with redox regulation—making interpretation of oxidative stress responses difficult. Nevertheless, the metabolic features of *rcd1* plants suggest that one of the many functions of RCD1 is to redirect the biosynthesis between different branches of phenylpropanoid pathway by favoring the lignin pathway over flavonoid production under control non-stressed conditions and by affecting the crosstalk between phenylpropanoid and GLS biosynthesis under stress potentially via its interaction with STO and COP1 to repress HY5. Moreover, RCD1 appears to inhibit the glycosylation of specialized metabolites in general. This coordination of carbon flow into the biosynthesis of specialized metabolites and the divergent regulation of different branches of phenylpropanoid biosynthetic pathway can also contribute to the frequently observed tradeoff between plant growth and stress tolerance [58,72]. Consistently elevated levels of phenolics such as hydroxycinnamic acids and flavonoids with profound ROS-scavenging and UV-absorbing activities likely underlie the several stress tolerance phenotypes of *rcd1* plants.

### 3.4. Low-Molecular Weight Antioxidant Pools Are More Efficiently Sustained in rcd1

**Figure 3:**
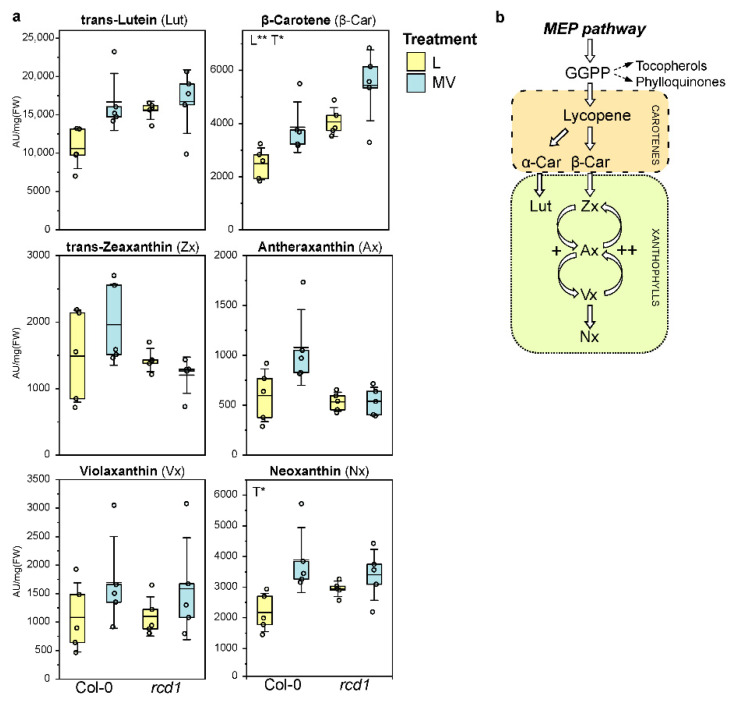
(**a**) Pigment levels (AU/mg[FW]) in Col-0 and *rcd1* plants in control/light conditions (L), and after paraquat exposure (MV), and (**b**) their biosynthesis pathway. Dots represent data points (*n* = 4–5); the box represents 25 and 75 percentiles; the line marks the mean and the whiskers the standard deviation. The main effects of plant line (L) and treatment (T) and their interaction (L × T) were tested with two-way ANOVA (FDR-adjusted *p*-value < 0.05 *, <0.01 **). + indicates epoxidation, ++ de-epoxidation reactions.

In plants, MV attracts electrons from PSI while inhibiting NADPH production and subsequently transferring them to molecular oxygen in MV redox cycle, resulting in the production of toxic ROS [1]. The *rcd1* plants are tolerant to MV, as previous studies have shown; the photosynthesis is already inhibited in Col-0 wild type, but not in *rcd*1 after MV exposure at the time point we sampled the plants for metabolomics [23]. Furthermore, previous studies indicate that MV resistance in *rcd1* is not due to altered transport of MV or restricted access of MV to PSI, but rather that inter-organellar communication plays a role in MV resistance [23,80]. Therefore, we studied how the levels of major small-molecular weight antioxidants, carotenoids, glutathione, ascorbate, and tocopherols, respond to MV exposure in Col-0 and *rcd1*. MV increases both the linear photosynthetic electron transport (ETR) and the pH gradient (∆pH) formation across the thylakoid membranes in chloroplasts, providing conditions for the de-epoxidation of the xanthophyll cycle. This leads to elevated production of zeaxanthin and antheraxanthin as well as to an increased de-epoxidation state (DEPS) [2,3,4,5]. However, the levels of xanthophyll cycle metabolites or the de-epoxidation state (DEPS) did not change significantly after 4h MV exposure in light in Col-0 or in *rcd1* (Figure 3, Supplementary Table S1l). This could be because de-epoxidation is reported to be time-, temperature-, and light-intensity-dependent, and the dark adaptation pre-treatment affects the response to MV in a photosynthetic system [2,3,4,5]. Zeaxanthin, which functions as an antioxidant and a fluorescence quencher, has been reported to accumulate rapidly after MV exposure as an early response, but the levels decline within hours depending on light and temperature [2,5,81,82].

**Figure 4:**
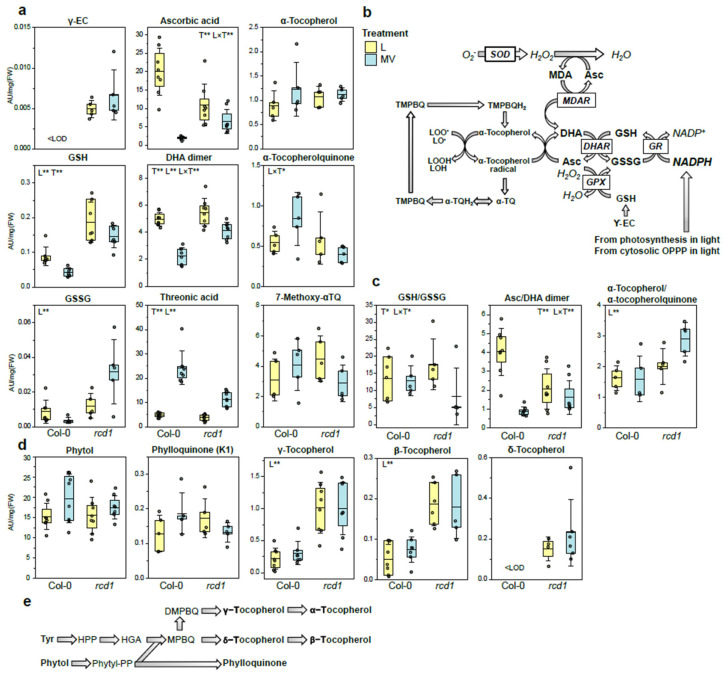
Metabolites involved in tocopherol metabolism and Foyer–Halliwell–Asada redox cycle. (**a**) Glutathione, ascorbate, and -tocopherol metabolite levels (AU/mg[FW]) in Col-0 and *rcd1* in control/light conditions (L), and after paraquat exposure (MV) and (**b**) simplified visualization of Foy-er–Halliwell–Asada cycle connected to tocopherol redox cycle. (**c**) Ratios between reduced and oxidized metabolite forms. (**d**) Phytol, phylloquinone, and tocopherol levels in Col-0 and *rcd1* in control/light conditions (L) and after paraquat exposure (MV) and (**e**) their biosynthesis pathway. Dots represent data points (*n* = 4–8), the box represents 25 and 75 percentiles; the line marks the mean and the whiskers standard deviation. The metabolite levels below limit of detection marked as <LOD. The main effects of plant line (L) and treatment (T) and their interaction (L × T) were tested with two-way ANOVA (FDR-adjusted *p*-value < 0.05 *, <0.01 **). Tocopherols (β-, γ-, δ-), phytol, and ascorbic acid metabolite levels were analyzed with GC-MS in Sipari et al. [13] and phylloquinone, glutathione, and α-tocopherol metabolite levels with LC-MS.

The most interesting differences between the plant lines’ responses to MV exposure were observed in the levels of glutathione, ascorbate, and tocopherols (Figure 4). Glutathione (GSH) biosynthesis is dependent on the levels of three amino acids: Cys, Glu, and Gly, while GSH/GSSG ratio is dependent on cellular redox state [36]. The levels of GSH as well as its precursor γ-glutamylcysteine (γ-EC) were significantly higher in *rcd1* than in Col-0 (γ-EC <LOD) irrespective of condition (in L and MV) (Figure 4a). The levels of oxidized form of glutathione (GSSG) were comparable in Col-0 and *rcd1* in control/light (L) conditions, but while the levels of GSH and GSSG decreased after MV exposure in Col-0, in *rcd1,* GSH levels remained high, and GSSG level tended to increase (Figure 4a, Supplementary Table S1b,d,h). This indicated that although GSH/GSSG ratio decreased (Figure 4a), the total glutathione pool was not depleted in *rcd1* as in Col-0 after MV exposure. Increased glutathione levels due to increased expression of cytosolic and/or plastidic glutathione reductases (GR) have been previously reported to increase the MV resistance in *Escherichia coli*, tobacco, and Arabidopsis [83,84,85,86], and both increased expression and abundance of GR enzymes in different cell compartments have similar effect to MV resistance [86]. The increased level of GSH and the regulation of the total glutathione pool in *rcd1* may have both direct effect to the cellular redox status, but it may also be alleviating MV-induced oxidative stress, which can be one factor behind MV resistance in *rcd1*.

In addition to altered glutathione levels, the responses of ascorbate metabolite levels to MV treatment differed between Col-0 and *rcd1.* As a general pattern, the levels of ascorbic acid (Asc), dehydroascorbic acid (DHA), and Asc/DHA ratio decreased after MV exposure, but much less in *rcd1* than in Col-0 (Figure 4). Although ascorbic acid level was lower in *rcd1* than in Col-0 in control/light conditions (L), it remained higher after MV treatment (Figure 4a). Similarly, DHA level and Asc/DHA ratio remained higher in *rcd1* than in Col-0 after MV treatment (Figure 4a). In both plant lines, the levels of the final Asc degradation product, threonic acid, increased after MV treatment, but this response was weaker in *rcd1* (2-fold) compared to Col-0 (4-fold) (Figure 4a). These results indicated that Asc pool and its redox state were more efficiently maintained in *rcd1* than in Col-0 under MV-induced oxidative stress but compromised under control conditions. Since ascorbate biosynthesis in mitochondria is linked to the ETC [38,39], the lower Asc levels and Asc/DHA ratio in *rcd1* than in Col-0 under control conditions could be linked to the altered mitochondrial functions which has been indicated by the elevated levels of metabolic markers and enhanced expression of MDS (mitochondrial dysfunction stimulon) genes [13,23].

The levels of the oxidized forms of α-tocopherol, α-tocopherol quinone (αTQ), and 7-methoxy-α-tocopherol, as well as the αT/αTQ ratio were similar between Col-0 and *rcd1* in control/light conditions (L), but the response to MV was opposite in the plant lines (Figure 4a,c). The levels of α-tocopherol did not change in response to MV in either plant line, but the level of α-tocopherol quinone (αTQ) increased in Col-0 while it decreased in *rcd1*, resulting in a higher α-T/αTQ ratio in *rcd1* than in Col-0 (Figure 4a,c). α-Tocopherol can physically quench singlet oxygen radicals and chemically scavenge singlet oxygen or lipid peroxyl radicals by producing tocopherol oxidation products [45,87]. α-Tocopherol quinone (αTQ) is produced during antioxidant activity from α-tocopherol radical, which can be recycled back to α-tocopherol by ascorbate and/or glutathione [88] connecting Asc, GSH, and tocopherol redox cycles (Figure 4b). In animal studies, the ratio of αTQ/αT is reported to increase (i.e., αT/αTQ to decrease) with increasing oxidative stress conditions [89]. In addition, the levels of three (β-, γ-, and δ-) out of four detected tocopherols were significantly higher in *rcd1* than in Col-0 irrespective of condition (Figure 4d).

Our study demonstrates that enhanced chloroplastic ROS production by MV can have different impact on the redox state of ascorbate and glutathione pools. This could be an outcome of differently localized pools; for example, the oxidized form of ascorbate, DHA, has been mainly found in the apoplast [36,90,91,92]. In the Foyer–Halliwell–Asada cycle, ascorbate is regenerated from DHA by GSH, either chemically or via DHARs [36] (Figure 4c). The altered status of both glutathione and ascorbate pools has been associated to enhanced ROS availability, and the Asc/DHA, GSH/GSSG, and αT/αTQ ratios have been reported to be shifted towards oxidized forms, and the ratio to decrease during (MV-induced) oxidative stress condition [3,36,89]. The changes in the levels of redox-cycling small-molecular-weight antioxidants indicate that not only the metabolite pools but also their redox state were more efficiently sustained in *rcd1* compared to Col-0, especially after MV exposure. In *rcd1*, the considerably higher abundance of glutathione and tocopherols can facilitate efficient regeneration of low-molecular-weight antioxidants and alleviate MV-induced oxidative stress. We propose that RCD1 acts as a focal point of regulation of antioxidant systems—under non-stressed conditions by inhibiting the biosynthesis of flavonoids, glutathione, and tocopherols and by sustaining the lignin pathway and its growth and the biosynthesis and redox state of ascorbate interlinked with mitochondrial ETC. Under oxidative stress, the disruption of RCD1 induces the biosynthesis of flavonoids and glutathione as well as tocopherols which are key antioxidants protecting cellular membranes from oxidative damage, while on the other hand the effects on mitochondrial functions can hinder ascorbate biosynthesis.

## 4. Conclusions

**Figure 5:**
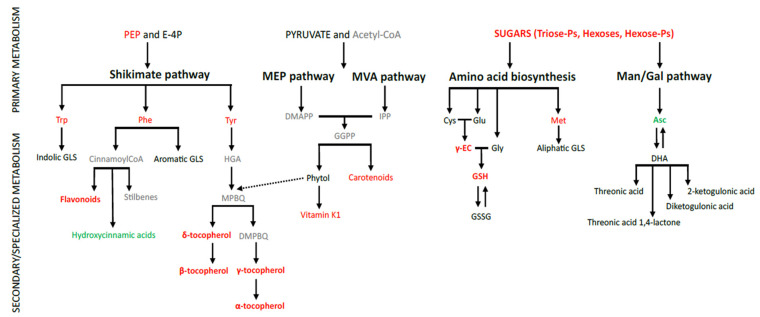
Simplified illustration of altered, specialized metabolic pathways by RCD1: carotenoid biosynthesis via MEP (methyl erythritol phosphate), MVA (mevalonate) pathways, shikimate pathway connected to aromatic amino acids, tocopherol and phylloquinone pathway, and aromatic and indole glucosinolates (GLS), as well as flavonoid, stilbene, and hydroxycinnamic acid (HCA) pathways. Black, red, and green colors indicate similar, increased, or decreased metabolite levels in *rcd1,* respectively. Grey metabolites were not detected. Amino acids (Phe, Tyr, Trp, and Met), phytol, ascorbate (Asc and DHA), and tocopherol levels measured previously with GCMS in Sipari et al. [13].

Methyl viologen (MV) resistance has been reported to be an outcome of enhanced sequestration or detoxification, its hindered transport, or enhanced ROS-scavenging ability by enzymatic antioxidants. In addition, enhanced expression of specific regulators (TFs) or genes connected to biosynthetic pathways and/or accumulation of various specialized secondary metabolites that have profound antioxidant capacities have been suggested to be linked to MV resistance. In our study, several metabolites connected to MV-resistance were consistently and significantly increased in *rcd1* plants, except levels of ascorbate, and several metabolites connected to sinapate and ester pathway were decreased significantly when compared to the wild type (Figure 5).

This indicates that RCD1 functions as an inhibitor of biosynthetic pathways producing several major specialized metabolite groups as well as tocopherols and glutathione that are key components of antioxidant system. On the contrary, RCD1 appears to sustain the ascorbate biosynthetic pathway inter-linked with the mitochondrial electron transport. RCD1 has been previously reported to act as an inhibitor of the oxidative stress responses via several interaction partners that are not only connected to various stresses but also to growth and development, as well as to mitochondrial dysfunction, and could be a systematic cause of MV resistance in *rcd1*. We propose that RCD1 acts as a focal point of redox regulation to maintain the basal antioxidant system under non-stressed conditions, and under oxidative stress, its disruption enhances antioxidant defense mechanisms.

## Data Availability

The normalized data analyzed with UPLC and GC−MS can be found in the Supplementary Table S1.

## Supplementary Materials

The following supporting information can be downloaded at: https://www.mdpi.com/article/10.3390/antiox11102034/s1. Additional Supporting Information may be found in the online version of this article: Supplementary Figure S1. Metabolite profiles in Arabidopsis as affected by line, light and methyl viologen treatments. Metabolites were analyzed by LC-MS with positive and negative ESI and the variation in metabolite profiles (1146 features) were studied with principal component analysis (PCA, a-c). In addition, the variation in lipophilic metabolite profile (16 metabolites analyzed with LC-MS using positive ESI) was studied (E). L = Light, MV = methyl viologen/paraquat. Supplementary Figure S2. Comparison of LC-MS metabolite levels between lines (a-b) and between treatments within each line (c–d) visualized with volcano plots. a) *rcd1*/Col-0 in light, b), *rcd1*/Col-0 in MV, C) MV/L in Col-0, and D) MV/L in *rcd1*. Red and blue colors indicate metabolites significantly (*p* < 0.05, FC > 1.2) increased or decreased between the comparisons, respectively. Supplementary Table S1a–l. S1a. Integrated peak areas, tentative identifications, references, chemical formulas, Exact masses and errors (mDa) between calculated and analyzed exact molecular ion masses of UPLC-ESI(-)/HDMS analysis normalized to ISTD and FW (g) in negative mode. S1b. Mean and standard error of metabolites in UPLC-ESI (-)/HDMS data, negative mode. S1c. Integrated peak areas, tentative identifications, references, chemical formulas, exact masses and errors (mDa) between calculated and analyzed exact molecular ion masses of UPLC-ESI(+)/HDMS analysis normalized to ISTD and FW (g) in positive mode. S1d. Mean and standard error of metabolites in UPLC-ESI (+)/HDMS data, positive mode. S1e. Integrated peak areas, identifications, chemical formulas, exact masses, retention times, UV maxima (nm), and errors (mDa) between calculated and analyzed exact molecular ion masses of UPLC-ESI (-)/HDMS analysis normalized to ISTD and FW (g) in positive mode. S1f. Mean and standard error of UPLC-PDA-ESI (+)/HDMS data in positive mode. S1g. Integrated peak areas of GC/MS analysis normalized to ISTD and FW (g). Original data in Sipari et al. [13]. S1h. Mean and standard error of GC/MS data. Original data in Sipari et al. [13]. S1i. 2-way ANOVA results of all data: GC/MS, UPLC-ESI/HDMS in positive (+) and negative (−) modes, and UPLC-PDA data of pigments. S1j. Pairwise comparisons for line effects in L and MV treatments and the effect of MV within each line in UPLC-MS data, tested with t-test (MetaboAnalyst). S1k. Pairwise comparisons for line effects in L and MV treatments and the effect of MV within each line in GC-MS data, tested with *t*-test (MetaboAnalyst). S1l. Pairwise comparisons for line effects in L and MV treatments and the effect of MV within each line in specific metabolite ratios, tested with *t*-test (MetaboAnalyst). References [93,94,95,96,97,98,99,100,101]

## Abbreviations

The following abbreviations are used in this manuscript:

1-MOI3M	1-methoxy-indolyl-3-methyl GLS
2-PE	2-phenylethyl GLS
4-MOI3M	4-methoxy-indolyl-3-methyl GLS
4-MSOB	4-methylsulfinylbutyl GLS
4-MTOB	4-methylthiobutyl GLS
4-OHI3M	4-hydroxy-indolyl-3-methyl GLS
5-MH	5-methylhexyl GLS
7-MSOH	7-methylsulfinylheptyl GLS
8-MSOO	8-methylsulfinyloctyl GLS
Ax	Antheraxanthin
AOX	Alternative Oxidase
Asc	Ascorbate, reduced
COX	Cytochrome C Oxidase
β-Car	β-carotene
DHA	Dihydroascorbate, oxidized
DHAR	Dihydroascorbate Reductase
DEPS	De-Epoxidation State
DMAPP	Dimethylallyl pyrophosphate
DMPBQ	2,3-Diethyl-6-Phytyl-1,4-Benzoquinol
GC-MS	Gas Chromatography—Mass Spectrometry
GGPP	Geranylgeranyl diphosphate
GLS	Glucosinolate
GR	Glutathione Reductase
GPX	Glutathione Peroxidase
GSH	Glutathione, reduced
GSSG	Glutathione, oxidized
HGA	Homogentisic Acid
HPP	p-hydroxyphenylpyruvate
I3M	Indolyl-3-methyl GLS
IPP	Isoprenyl pyrophosphate
LOD	Limit Of Detection
LOH/LOOH	Lipid hydroxides/hydroperoxides
Lut	Lutein
MDA	Monodehydroascorbate, oxidized
MDAR	Monodehydroascorbate Reductase
MDS	Mitochondrion Dysfunction Stimulon
MEP	Methyl-erythritol-4-phosphate
MPBQ	2-Methyl-6-Phytyl-1,4-Benzoquinol
MV	Methyl viologen, paraquat
Nx	Neoxanthin
PCA	Principal Component Analysis
Phytyl-PP	Phytyl diphosphate
RCD1	Radical-induced Cell Death1
ROS	Reactive Oxygen Species
α-T	α-tocopherol
α-TQ	α-tocopherolquinone
TQH2	Tocopherolhydroquinone
TMPBQ	2,3,5-trimethyl-6-phytyl-1,4-benzoquinone
TMPBQH2	2,3,5-trimethyl-6-phytyl-1,4-benzoquinol
UPLC-MS	Ultrahigh-performance Liquid Chromatography—Mass Spectrometry
Vx	Violaxanthin
Zx	Zeaxanthin
