# Supplementary material for: Metabolite Profiling of Paraquat Tolerant Arabidopsis thaliana Radical-induced Cell Death1 (rcd1)—A Mediator of Antioxidant Defence Mechanisms
Source: Antioxidants (Basel). 2022 Oct 15;11(10):2034. doi: 10.3390/antiox11102034 (PMC9598866; doi:10.3390/antiox11102034)
Supplement: Supplementary file 1 [file antioxidants-11-02034-s001.zip › Supplementary Figures.pdf]

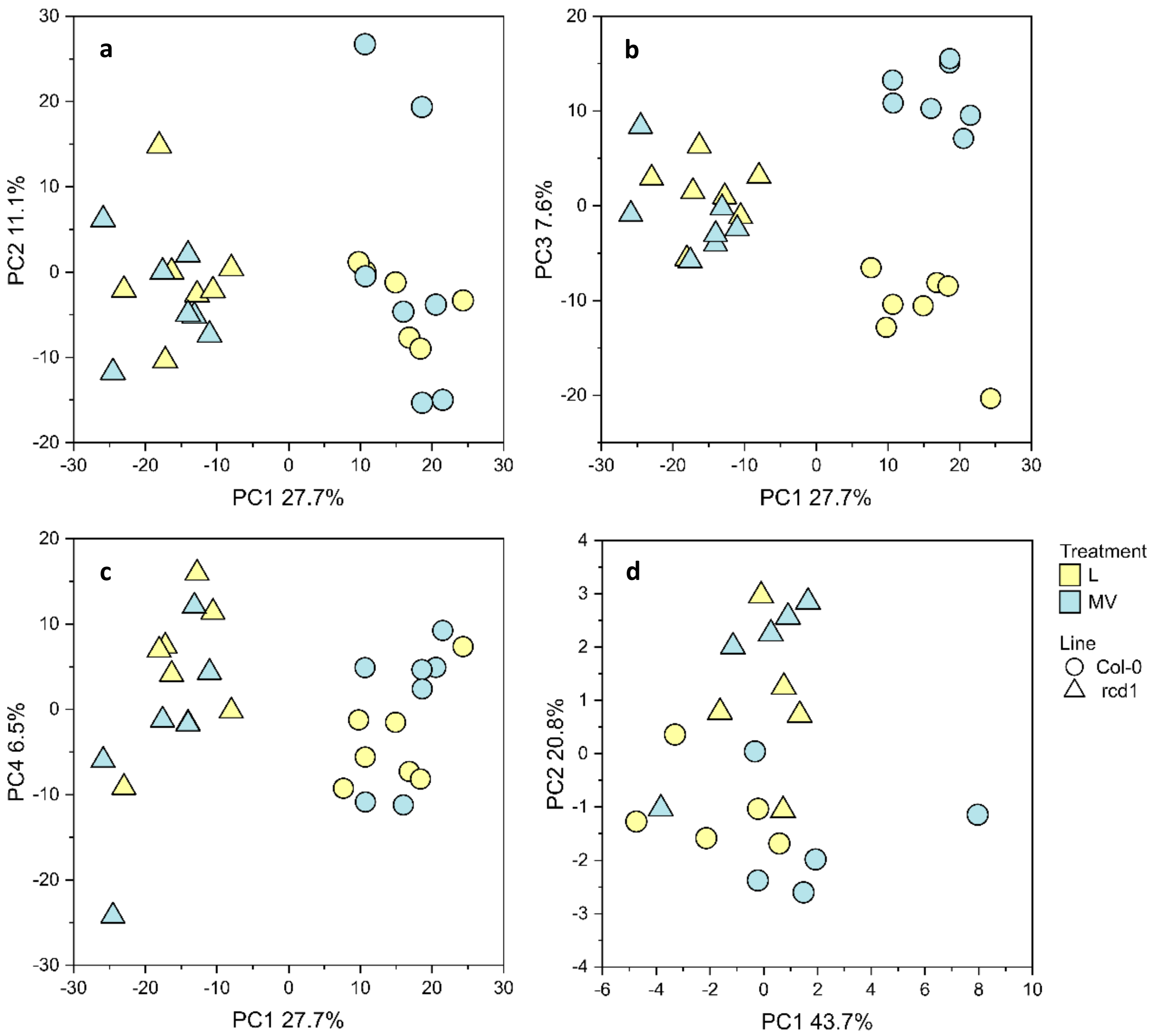

Supplementary Figure S1. Metabolite profiles in Arabidopsis as affected by line and light and methyl viologen treatments. Metabolites were analysed by LC-MS with positive and negative ESI and the variation in metabolite profiles (1146 features) were studied with principal component analysis (PCA, **a-c**). In addition, the variation in lipophilic metabolite profile (16 metabolites analysed with LC-MS using positive ESI and PDA) was studied (**d**). L=Light, MV=methyl viologen/paraquat.

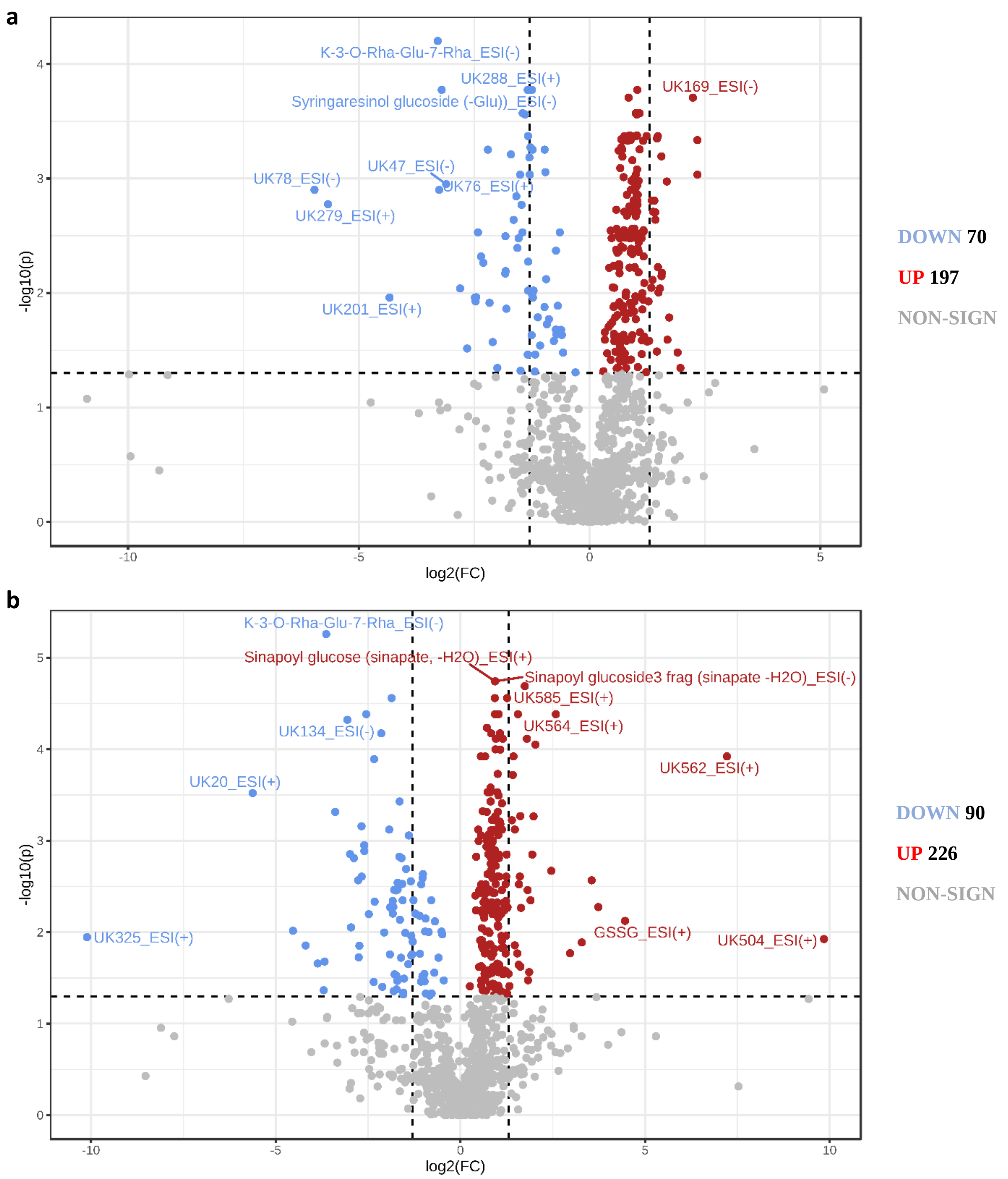
a
K-3-O-Rha-Glu-7-Rha_ESI(-)
UK288_ESI(+)
Syringaresinol glucoside (-Glu))_ESI(-)
UK169_ESI(-)
UK47_ESI(-)
UK78_ESI(-)
UK76_ESI(+)
UK279_ESI(+)
UK201_ESI(+)
-log10(p)
log2(FC)
DOWN 70
UP 197
NON-SIGN
b
K-3-O-Rha-Glu-7-Rha_ESI(-)
Sinapoyl glucose (sinapate, -H2O)_ESI(+)
Sinapoyl glucoside3 frag (sinapate -H2O)_ESI(-)
UK585_ESI(+)
UK134_ESI(-)
UK564_ESI(+)
UK20_ESI(+)
UK562_ESI(+)
UK325_ESI(+)
GSSG_ESI(+)
UK504_ESI(+)
-log10(p)
log2(FC)
DOWN 90
UP 226
NON-SIGN

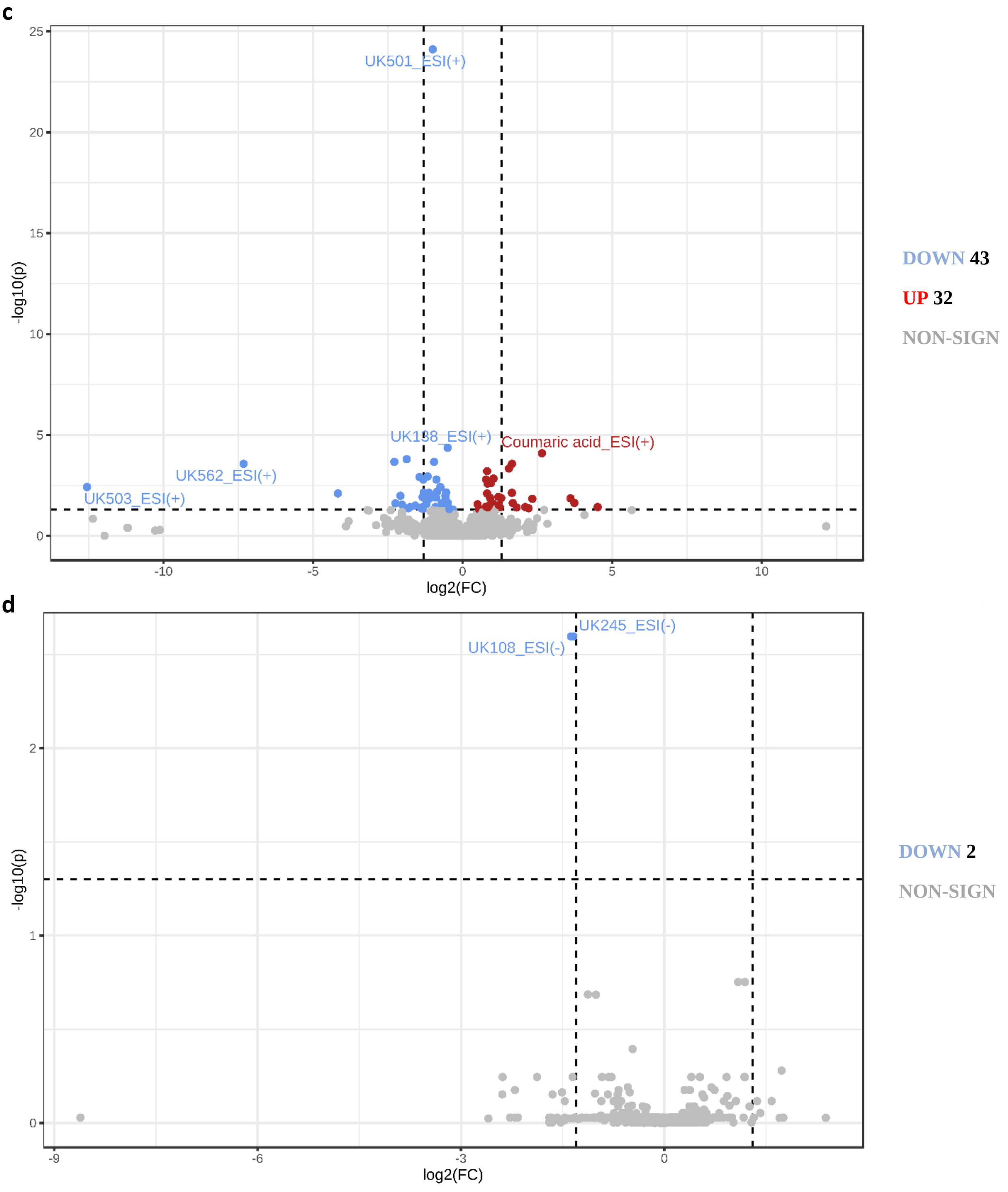

Supplementary Figure S2. Comparison of LC-MS metabolite levels between lines (a-b) and between treatments within each line (c-d). **a**) *rcd1*/Col-0 in light, **b**), *rcd1*/Col-0 in MV, **c**) MV/L in Col-0, and **d**) MV/L in *rcd1*.
